# Effects of general practitioner training and family support services on the care of home-dwelling dementia patients - Results of a controlled cluster-randomized study

**DOI:** 10.1186/1472-6963-10-314

**Published:** 2010-11-18

**Authors:** Carolin Donath, Elmar Gräßel, Maria Großfeld-Schmitz, Petra Menn, Jörg Lauterberg, Sonja Wunder, Peter Marx, Stephan Ruckdäschel, Hilmar Mehlig, Rolf Holle

**Affiliations:** 1 Psychiatric University Clinic Erlangen, Schwabachanlage 6, 91054 Erlangen, Germany; 2 Helmholtz Zentrum München, Ingolstädter Landstr. 1, 85764 Neuherberg, Germany; 3 Federal Association of the AOK, Rosenthalerstr. 31, 10178 Berlin, Germany; 4 AOK Bavaria - Health insurer, Stromerstraße 5, 90443 Nürnberg, Germany; 5 Pfizer Deutschland GmbH, Linkstrasse 10, 10785 Berlin, Germany; 6 HealthEcon AG, Basel, Switzerland; 7 Eisai GmbH, Frankfurt, Germany

## Abstract

**Background:**

More than 90% of dementia patients are cared for by their general practitioners, who are decisively involved in the diagnosis, therapy and recommendation of support services. *Objective: *To test whether special training of general practitioners alters the care of dementia patients through their systematic recommendation of caregiver counseling and support groups.

**Method:**

129 general practitioners enrolled 390 dementia patients and their informal caregivers in a prospective, three-arm cluster-randomized 2-year study. Arm A constituted usual care, in Arm B and C support groups and caregiver counseling (in Arm B one year after baseline, in Arm C at baseline) were recommended by the general practitioners. The general practitioners received arm-specific training. Diagnostic and therapeutic behavior of physicians was recorded at baseline. Informal caregivers were questioned in follow-up after 2 years about the utilization of support services.

**Results:**

The diagnostic behavior of the general practitioners conforms to relevant guidelines. The procedure in newly-diagnosed patients does not differ from previously diagnosed patients with the exception of the rate of referral to a specialist. About one-third of the newly-diagnosed dementia patients are given an anti-dementia drug. The utilization of support groups and counseling increased five- and fourfold, respectively. Utilization of other support services remained low (< 10%), with the exception of home nursing and institutional short-term nursing.

**Conclusion:**

Trained general practitioners usually act in conformity with guidelines with respect to diagnosing dementia, and partly in conformity with the guidelines with respect to recommended drug therapy. Recommendations of support services for informal caregivers by the general practitioner are successful. They result in a marked increase in the utilization rate for the recommended services compared to offers which are not recommended by the general practitioner.

**Trial registration:**

ISRCTN68329593

## Background

Medical care of elderly people in Germany is given by general practitioners in the majority of cases. This applies also to dementia patients: more than 90% are diagnosed and cared for by the general practitioner [1]. He is responsible for the initiation of the diagnostic process, the individual combination of pharmaceutical or non-pharmaceutical options and consultation with informal caregivers regarding support services available near the place of residence. The majority of general practitioners has a clearly positive attitude toward the treatment of dementia patients [2], but is well-aware of the difficulties [3,4], such as communicating the diagnosis and the subsequent conse-quences [5,6].

### Diagnostics and Therapy - Status quo

Various studies have thus far investigated the diagnostic behavior of general practitioners toward dementia patients [1,7-9]. Stoppe et al. [10] found that general practitioners do not differ from specialists in Neurology/Psychiatry in their early-diagnostic attention to Alzheimer's disease. Nonetheless, many general practitioners want to learn more about dementia illnesses [3], especially about diagnostic methods and management of behavior impairments [9]. The same results were also shown in other British and Scandinavian studies [11,12]. Neuropsychological test procedures are seldom used in family practice because of the time required [13,14]. The necessity of performing at least brief screening tests like the MMSE has been increasingly recognized in recent years by general practitioners [8]. Still, there is frequent criticism that only a small number of dementia patients undergo appropriate diagnostics and receive appropriate therapy [7,8,14]. This usually happens due to a lack of knowledge, but often also because the diagnosis is taboo, the therapeutic options are apparently limited, or because it is feared that the doctor-patient relationship will suffer by bringing bad news.

In the literature, a guideline-supported care rate of patients with Alzheimer's disease (AD) with evidence-based anti-dementia drugs of 26% [15,16] or even less [17] is reported for Germany, although the majority of general practitioners is convinced of at least a moderate effect of anti-dementia drugs [18]. In other west-European countries, the therapy rate is considerably higher: 53% in Spain [19], 46% in Ireland, 47% in Sweden and 50% in France and Finland [16,20]. In east-European countries, the treatment rate is considerably lower, for example in the Czech Republic 9% and Bulgaria 6% [16]. Care rates with anti-dementia drugs for patients with AD similar to that in Germany are found in Switzerland and in Denmark (each 28%) and in Great Britain (18%). US-data show that in the population of home-dwelling dementia patients, who are investigated in our study, the therapeutic rate is 27% [21].

### Guidelines on Diagnostics and Therapy

For diagnostics, the International Guideline of the National Institutes for Health and Clinical Excellence (NICE) recommend thorough exploration, including the medical history, a physical-clinical examination, and performance of a cognitive screening. Moreover, a laboratory series (blood count, biochemical parameters, thyroid function, Vitamin B12 and folic acid) should be part of the basic screening. Other procedures (urine, X-ray, ECG) should only be performed after careful consideration. Imaging procedures are recommended for diagnostics of dementia subtypes [22]. The diagnostic measures recommended in the Dementia Guidelines developed for general practitioners by the German Society for General Medicine [23] include the history (self and wherever possible third-party), cognitive performance tests (screening) and physical examination, as well as laboratory diagnostics and, in the case of unclear findings, imaging and referral to a specialist. The American Guideline of the Psychiatric Association does not differ in its diagnostic recommendations from the procedures cited but emphasizes that imaging procedures would be desirable for all patients. This is, however, especially important if the disease begins early (at less than 65 years of age) and with respect to vascular risk factors [24].

For the treatment of AD patients, the German "Dementia" Guideline for general practitioners recommends drug therapy (cholinesterase inhibitors, Memantine) and non-drug therapy (e.g. psychomotor activation, cognitive stimulation), whereby the use of anti-dementia drugs is subject to critical discussion [23]. The critical point of view is confirmed in the British Guideline, which recommends treatment with cholinesterase inhibitors only in the moderate stage (with justified exceptions) and argues against the use of Memantine except in clinical studies [22]. The US-American Guideline by the Psychiatrists clearly favors the treatment of AD patients with evidence-based anti-dementia drugs (cholinesterase inhibitors, Memantine). It recommends cholinesterase inhibitors for mild and moderate stages of AD and does not rule out a benefit for severely demented AD patients [24]. In Germany, by contrast, cholinesterase inhibitors have been approved only for mild and moderate AD and this use is also included in the Guidelines for general practitioners and specialists [25]. Both the German and the American Guidelines describe moderate and severe AD as the area of use for Memantine. None of the Guidelines cited contains a clear recommendation for the use of cholinesterase inhibitors or Memantine in purely vascular dementia.

### Guidelines for Support of Informal caregivers

In addition to treating dementia patients, the aim is to support informal caregivers with appropriate support and respite services which have been found to be effective [26-28], such as caregiver counseling or caregiver support groups. If a informal caregiver experiences less stress in caring, the patient usually can remain longer in his familiar environment [29]. This corresponds to the wishes of most of the patients and their families, and would also relieve the cost burden on health care systems. The Guideline "Informal Caregivers" published by the German Society for General Medicine [30] defines a counseling obligation for the family practitioner with respect to support services for informal caregivers. The international guidelines in the UK and the USA also recommend counseling and support services for informal caregivers ("peer-support groups, information services, psychoeducation" etc.), which must be mediated by the treating physician [22,24]. However, the coordination of support services is viewed by general practitioners as one of the greatest difficulties in treating dementia patients [9]. Usually the general practitioner has too little knowledge of respite and support services near the place of residence [1,31] such as counseling for informal caregivers and support groups, but also concerning training courses in care, day care centers, day clinics, home nursing and home help services, institutional short-term nursing or voluntary helpers. Sometimes there actually are gaps in the services regionally available [32].

### Objective

In the German IDA-Study (**I**nitiative **D**emenzversorgung in der **A**llgemeinmedizin: Dementia Care Initiative in Primary Practice) presented here, the care of dementia patients living at home and their informal caregivers was to be optimized with respect to diagnostics, therapy and support services. This took place on the basis of training of general practitioners and systematic recommendation of support groups in the form of peer groups and actively approaching caregiver counseling of informal caregivers.

The objective of this paper is to examine whether a change in the care process took place. The individual questions addressed are:

Is it possible to change the care of patients with respect to diagnostics and drug therapy by means of training the general practitioners?

To what extent can the utilization of support services (caregiver counseling and support groups) be increased by recommendation of the general prac-titioner?

What effect does the utilization of family caregiver counseling have on the utilization of other available support services?

## Method

### Design

This was a three-armed cluster-randomized controlled longitudinal study of patients with dementia living at home with at least one care-giving informal caregiver. Patients in the study region of Middle Franconia, Bavaria, Germany, were included if they had mild or moderate dementia (MMSE 10-24) according to the diagnosis criteria of DSM-IV [33] respectively ICD-10 [34], were at least 65 years old and were members of the AOK Bavaria - Health insurer. General practitioners have been trained in the diagnostic criteria of ICD-10 and DSM-IV and in applying the MMSE. When diagnosing the patients and checking for inclusion criteria, there was in the first step the MMSE applied and additionally checked - if the MMSE was between 10 and 24 points - if the criteria of the DSM-IV respectively ICD-10 for dementia were fulfilled. Study recruitment and follow-up took place from July 2005 to January 2009. The patients were enrolled in the study by their general practitioners. Follow-up examinations were conducted after 1 and 2 years in patients still living at home.

General practitioners in all study groups participated in a training course on dementia diagnosis. In the two intervention groups B and C, they additionally received training on evidence based dementia treatment. They also recommended support groups and actively approaching family counselling to caregivers beginning either at baseline (study Arm C) or after the 1-year follow-up (study Arm B).

The study was approved by the Ethics Committee of the Bavarian Chamber of Physicians (No. 05029, date of approval: 30.05.2005). Both the patients and their informal caregivers were required to sign a written informed consent to participate in the study. The study is registered under the number ISRCTN68329593 at http://www.controlled-trials.com/. A detailed description of the study design has already been published elsewhere [35].

### Interventions

#### Training of General Practitioners

Training was held based on the current guidelines at the start of the study. The basic knowledge sources were the Guideline for Diagnostics and Treatment of Dementia for General Practitioners, published by the University of Witten Herdecke under http://www.evidence.de[36,37], the recommendations for drug therapy of the Drug Commission of the German Medical Council [38] and the Guideline "Informal Caregivers" by the German Society of General Medicine [30]. The dementia-specific contents were taught by five practicing neurologists and psychiatrists with geriatric-psychiatric expertise. Further details on training have already been reported elsewhere [39]. The doctors remained free in their diagnostic and therapeutic decision after training, despite participating in the study. This was intended to simulate real life medical care and thus to enhance generalization of study findings.

#### Training of General Practitioners - Basic training

All participating general practitioners underwent basic training. In addition to the study procedures and documentation of the study data, dementia-specific contents were included in the training curriculum. During the basic training, the physicians did not yet know to which study arm they had been randomized. The dementia-specific segment lasted 180 minutes and covered dementia epidemiology, etiology and knowledge and capability for dementia diagnostics: early symptoms, physical ex-amination, laboratory diagnostics, imaging and screening test (MMSE by means of lecture, case examples and self-performance by means of video demonstration).

#### Training of General Practitioners - Additional training (only Arms B and C)

The doctors in Arms B and C received an additional 140-minute unit of training. This addressed drug and non-drug therapy possibilities, and available care possibilities and services under the German Health Care system. The doctors were trained with respect to information and counseling of the informal caregivers of dementia patients.

#### Recommendation of Caregiver Support Groups

In Arms B and C, the doctor recommended that the informal caregiver(s) attend a caregiver support group. The informal caregiver was given a list of contact data for 18 support groups in the study region. These groups had to be conducted by a specialist, contain psycho-educative elements and had to take place at least 10 times per year. Whether the dementia patients' informal caregivers actually did attend a group remained solely their personal decision, again in order to have a naturalistic scenario.

#### Recommendation of Caregiver Counseling

In Arm C, actively approaching family counseling was recommended by the general practitioners at baseline, in Arm B after 1 year. Actively approaching counseling means that, after enrolment in the study, a trained counselor contacted the informal caregiver by telephone. The objective was to establish at least one personal contact (home visit). Starting from this contact, which was used for a needs assessment of support of the patient and more so of the informal caregiver, other counseling activities were undertaken. They followed a Case/Care Management approach. That means that information was provided case-specifically about further formal support services, such as nursing services, voluntary help etc. If desired, dementia-specific knowledge was provided and emotionally-relieving discussions held. The counseling was performed in the study region by four counselors with geriatric and psychiatric expertise and several years of experience in care and counseling. The concept of actively approaching caregiver counseling is described in detail elsewhere [40].

### Participants

**Figure 1:**
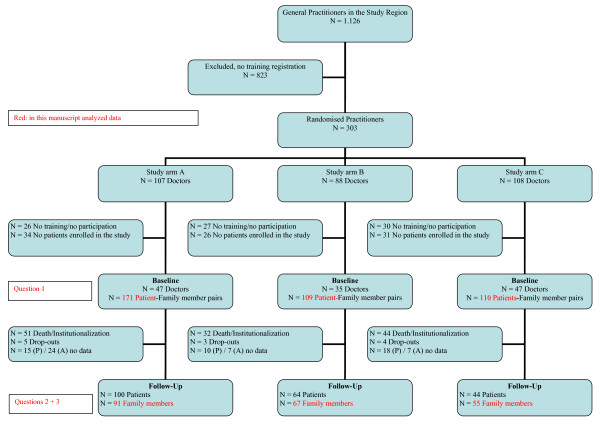
**Randomization, Recruitment of Participants and Drop-Outs**.

303 general practitioners in Middle Franconia were randomized (ca. ¼ of all practicing general practitioners). Of these, 129 practitioners enrolled 390 patients in the study, and 357 informal caregivers of these patients were questioned. After 2 years, 213 informal caregivers could still be contacted. 47 patients had entered a nursing home and 80 patients had died. The course of recruitment, randomization and the ratio between the drop-outs and follow-up samples are shown as a flow chart in Figure 1.

The characteristics of participating patients and informal caregivers are shown in Table 1.

**Table 1:** Description of the Sample

	Patients (n = 390)	Informal caregivers (n = 357)
Age [m (s)]	80.3 (6.8)	59.4 (13.4)

Sex (% female)	68.2	73.3

Relationship (%)		
• Marital partner		32.2
• Children/Children-in-law		59.1
• Other		8.7

Employed (%)	-	35.5

Shared living quarters (%)	68.6	68.6

Residence urban (> 100000 residents) (%)	24.4	
Residence rural (< 100000 residents) (%)	75.6	

MMSE [m (s)]	18.7 (3.8)	-
Mild dementia (18 - 24 points) (%)	65.1	
Moderate dementia (10 - 17 points) (%)	34.9	

Care level^§ ^(%):		
• none	57.4	
• 1	21.8	
• 2	18.0	
• 3	2.8	

Type of dementia (%)		
• Alzheimer Type	37.2	
• Vascular Dementia	27.4	
• Mixed form	5.1	
• not more precisely listed	30.3	

Barthel-Index [m (s)]	72.9 (26.5)	

^§ ^Social care insurance = legal obligatory insurance for all non-self-employed workers, pays money and material help to people needing care. Need for care is estimated by experts: Level 1 means needs help for at least 90 min/day; Level 2 daily at least 3 h; Level 3 daily at least 4 h in performing activities of daily living (ADL) and household tasks.

### Instruments

*For Question 1*: In addition to the questions and instruments for sample description (see Table 1), the diagnostic behavior of the general practitio-ners with respect to physical examination, laboratory diagnostics, imaging and referral to a specialist were addressed with two answer possibilities: "already performed" (yes/no) and "ordered"(yes/no). The frequency of use of screening tests could not be evaluated, since the MMSE had to be performed to examine the inclusion criteria. Thus, the screening rate was 100%. The drug-therapeutic behavior of the general practitioners was examined with the question whether anti-dementia drugs were currently being used and if so, which drug(s). Only anti-dementia drugs approved specifically for the diagnosis were considered: Donepezil, Galantamin, Rivastigmin (approved in Germany for mild to moderate Alzheimer's dementia) and Memantine (approved in Germany for moderate to severe Alzheimer's dementia).

*For Question 2: *The informal caregivers were asked in the 2-year follow-up whether they had contact during the past 2 years with a caregiver counselor or a support group, and if so, to what extent.

*For Question 3: *The informal caregivers were asked at the beginning of the study and at the 2-year follow-up whether they had utilized the following additional formal support services during the last 4 weeks: home nursing, home help, meals on wheels, daycare, day clinic, care groups, visiting voluntary helpers (in-home respite), institutional short-term nursing (within the past 12 months) and also whether the patient had received outpatient occupational therapy or outpatient physiotherapy within the last 4 weeks. The assessment was performed specific to the study arm, and as an urban/rural comparison. Urban was defined as a city with 100,000 residents or more.

### Assessment and Statistical Analyses

Assessment was made on the basis of complete cases. For Question 1 there are no completely missing cases, since the question addresses baseline-data. A few missings at the item level concerning the question whether diagnostic procedures were performed or a drug prescribed were conservatively entered with "not performed" or "not prescribed". Questions 2 and 3 addressed data of utilization which were answered with yes or no. Here, too, a few missing entries were entered as "not used". The statistical assessment was performed with SPSS 17.0.

Differences in nominal-scaled data were examined with the Chi²-Test. In Questions 1b, 2 and 3, differences between the study arms were examined, since the training contents and the recommended services differed. No check between the study arms was made in Question 1a (diagnostics), since practitioners in all study arms were trained in diagnostic procedures. Instead, a check was made whether the diagnostic behavior toward newly-diagnosed patients ("new diagnosis") differed from earlier diagnostic behavior (measures which had been performed in "old diagnoses"). In Question 1b (therapeutic behavior), the significance values were adjusted for cluster-effects. A fundamental assumption of the standard statistical methods used to analyse patient randomised trials is that the outcome for an individual patient is completely unrelated to that for any other patient. They are said to be "independent". This assumption is violated, however, in cluster trials because patients within any one cluster are more likely to respond in a similar manner. Cluster trials that do not account for clustering during analysis have artificially extreme p-values and overly narrow confidence intervals increasing the chances of spuriously significant findings and misleading conclusions. Therefore, the intra-class correction (ICC) was applied for this possibly distorting effect. The adjustment procedure is described by Donner [41]. The uncorrected p-values were used for the question of comparison of diagnostic behavior between new and old diagnoses, since both groups consist of patients in one cluster making the intra-class correlation less important. For Question 3, significance in univariate analysis in the differences in utilization of support services was additionally checked in multiple logistic regression taking control variables into account. The dependent variable is then utilization of each support offer. Controlled were the patient's disability level (Barthel-Index), study arm and urban versus rural area. The significance level for all analyses was set to α < .05. The sample of contacted informal caregivers after 2 years (no death or institutionalization of the patient during the observation period) served as the basis for determining the percentages in Questions 2 and 3. Informal caregivers, whose relative had died or entered a nursing home during the 2-year period were thus in the study for less than 2 years and had already had a higher utilization rate at baseline for all services than the complete cases. Taking all baseline values into account would thus signify distortion or impossibility of interpreting changes between baseline and follow-up.

## Results

### Diagnostic Procedure

New diagnoses of dementia (incident cases) were made in 31% of the patients within the framework of the study. The diagnosis had already been made in two-thirds of the patients enrolled. The diagnostic procedure is described below separately for "new" or "old" diagnosis (Table 2).

**Table 2:** Diagnostic Behavior of General practitioners

Measure	Newly diagnosed cases (n = 121)	Previously diagnosed cases (n = 269)	p-value*
Physical examination	104 (86.0%)	performed: 231 (85.9%)	.675^1^
		ordered: 6 (2.2 %)	

Imaging procedure	52 (43.0%)	performed: 121 (45.0%)	.264^2^
		ordered: 7 (2.6%)	

Laboratory diagnostics	116 (95.9%)	performed: 245 (91.1%)	.548^3^
		ordered: 17 (6.3%)	

Referral to a specialist because of dementia	55 (45.5%)	performed: 166 (61.7%)	**< .0001**^4^
		ordered: 5 (1.9%)	

* Chi²-Test

^1 ^Chi² (2) = .787

^2 ^Chi² (2) = 2.665

^3 ^Chi² (2) = 1.203

^4^Chi² (2) = 24.691

The highest rates with more than 85% were recorded for measures of physical examination and laboratory diagnostics. About half of the patients underwent an imaging procedure (cCT or cMRT). In these three diagnostic categories, the practitioners do not differ between their present procedure with newly-diagnosed patients after training and their earlier procedure. The only difference is that newly-diagnosed patients were significantly less often referred to a specialist because of dementia at the time of recording.

### Drug Therapy

About one-third (33.9%) of the 121 newly-diagnosed dementia patients were treated with a specific anti-dementia drug independent of accordance with the guideline. Although descriptively somewhat more patients in Arms B and C received drug therapy (37% instead of 29%), there is no statistically significant difference between the study arms (Table 3). To check therapy conformity with the guidelines, an analysis was made of the number of first-diagnosis patients with purely vascular-dementia (N = 30) treated with an anti-dementia drug which is not approved for that indication (Cholinesterase-inhibitor or Memantine). In Arms B and C, in which the practitioners were trained in therapy, this value was lower with marginal significance (p = .058). Moreover, a check was made with respect to conformity with guidelines whether drug-treated, first-diagnosis patients with mild dementia (N = 32) were treated with Memantine, which is approved only for moderate and severe Alzheimer's dementia. The result shows no statistically-significant group difference, whereby descriptively treatment of the patients especially in Arm B, but also in Arm C, was more in conformity with guidelines than in Arm A, in which practitioners were not trained in this respect (p = .326).

**Table 3:** Drug therapy of newly-diagnosed dementia patients by the family practitioner

First diagnoses	A (n = 48)	B (n = 41)	C (n = 32)	p-value*	Adjusted p-value
Prescription of specific anti-dementia drug (independent of guideline)^§^	14 (29.2%)	15 (36.6%)	12 (37.5%)	.671^1^	.857

**First diagnoses with purely vascular dementia**	A (n = 10)	B (n = 10)	C (n = 10)		

No cholinesterase inhibitor/Memantine in purely vascular dementia (in conformity with guideline)	4 (40.0%)	7 (70.0%)	9 (90.0%)	.058^2^	.071

**First diagnoses with mild dementia with specific anti-dementia treatment**	A (n = 11)	B (n = 12)	C (n = 9)		

No Memantine in mild dementia (in conformity with guideline)	6 (54.5%)	10 (83.3%)	6 (66.7%)	.326^3^	.477

^§ ^cholinesterase inhibitor/Memantine

* Chi²-Test

^1 ^Chi² (2) = .797

^2 ^Chi² (2) = 5.700

^3 ^Chi² (2) = 2.239

### Support services: Support Groups and Caregiver Counseling

**Figure 2:**
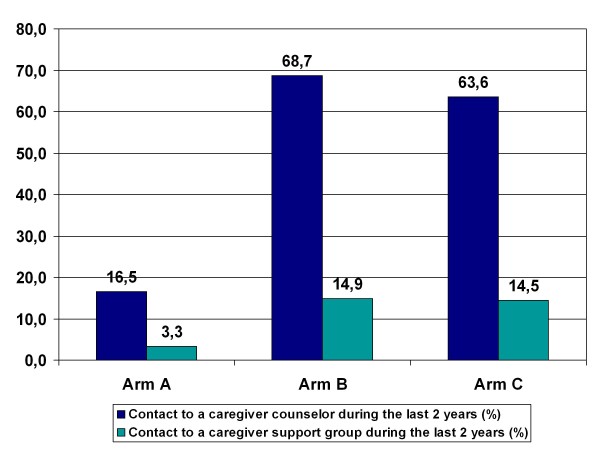
**Utilization rates of Support Groups and Caregiver Counseling at follow-up**.

Utilization rates of support services by the informal caregivers could be successfully increased by general practitioner's systematic recommendation. The utilization of caregiver counseling did not differ significantly at baseline (p = .986) between the study arms and was at a very low level (less than 3%). Likewise, the support groups were utilized by only 2% of informal caregivers at the start of the study, independent of the study arm (p = 1.000). There was a significant difference between the study arms concerning the informal caregivers who were still in the study after 2 years (the patient had not entered a nursing home or died) (N = 213), in their utilization of support groups (p = .021) and caregiver counseling (p < .0001) within the last 2 years. In Arm B and Arm C, where the services were recommended by the general practitioner at the start of the study respectively after one year, the utilization rates were 4 to 5 times higher than in the control group A (Figure 2).

### Utilization of further Support Services

Before the utilization of support and relief services was analyzed, a check was made to determine whether the percentage of patients with no assigned care level (impairment level following the formal graduation of the German Social Code Book XI) was the same in all three study arms. It is known that the assignment of a care level and the attendant payment of funds from social care insurance increases the utilization of support services [42]. The percentage of patients with no assignment of a formal care level is between 51% and 61% and does not differ significantly between the study arms (p = .736).

**Figure 3:**
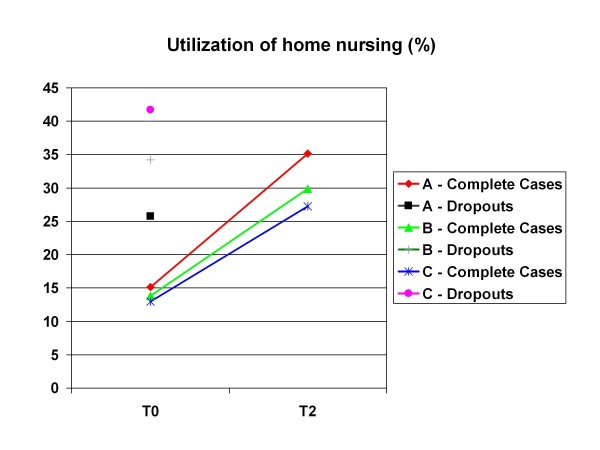
**Differences in the utilization of Home Nursing between Drop-Outs and Complete Cases at baseline**.

Only informal caregivers of patients who were still in the study at follow-up were included (N = 213). The reasons were presented above in the section on Assessment and Statistical Analyses. Using home nursing as an example, Figure 3 shows the differences in utilization between drop-outs and complete cases which already existed at the start of the study.

The utilization rates for 10 family and patient-oriented services at baseline and follow-up are presented in Table 4. In addition, the table contains the utilization rates in urban and rural areas. There, too, is no statistically significant difference in patient characteristics (percentage without an assigned care level) (p = .373). At the start of the study, there were almost no differences in the utilization of support services between the study arms and between urban and rural residence. Only outpatient physiotherapy was more often used by patients in the study Arm A. Multivariate analysis with control of the Barthel-Index (measure of independence in the area of ADL) confirmed this significant difference between the study arms at baseline. At the time of follow-up, there were no significant differences in utilization between the study arms and thus no higher use of support or respite services in the intervention Arms B and C. Moreover, the utilization rates did not differ between urban and rural residence. The use of support and respite services - with the exception of home nursing and institutional short-term nursing - remained at a very low level (less than 10%). Group-independent significant changes in utilization over time during the two years in the sense of increased utilization were observed only for home nursing (p < .0001), institutional short-term nursing (p < .0001), daycare (p = .006) and meals on wheels (p = .049) - but this increase is group-specifically significant only for home nursing (in all three arms) and institutional short-term nursing (in Arms A and B).

**Table 4:** Utilization of additional Support and Relief Services: Study arm and urban/rural comparison (N_baseline _= 205*; N_follow-u__p _= 213)

Offer	T	A N_T0 _= 86 N_T2 _= 91	B N_T0 _= 54 N_T2 _= 67	C N_T0 _= 65 N_T2 _= 55	p-Value Group differences	p < .05 between T_0 _und T_2_	Urban N_T0 _= 42 N_T2 _= 45	Rural N_T0 _= 163 N_T2 _= 168	p-Value Urban/Rural
Home nursing^1^	T_0_	13 (15.1%)	9 (13.8%)	7 (13.0%)	.935	A, B, C	9 (21.4%)	20 (12.3%)	.129
	T_2_	32 (35.2%)	20 (29.9%)	15 (27.3%)	.575		12 (26.7%)	55 (32.7%)	.436

Home help^1^	T_0_	4 (4.7%)	4 (6.2%)	2 (3.7%)	.820	-	3 (7.1%)	7 (4.3%)	.445
	T_2_	5 (5.5%)	6 (9.0%)	5 (9.1%)	.625		4 (8.9%)	12 (7.1%)	.693

Visiting voluntary helpers^1^	T_0_	1 (1.2%)	0	1 (1.9%)	.577	-	0	2 (1.2%)	.471
	T_2_	2 (2.2%)	4 (6.0%)	2 (3.6%)	.467		3 (6.7%)	5 (3.0%)	.248

Care group^1^	T_0_	1 (1.2%)	1 (1.5%)	1 (1.9%)	.945	-	1 (2.4%)	2 (1.2%)	.579
	T_2_	4 (4.4%)	3 (4.5%)	1 (1.8%)	.680		1 (2.2%)	7 (4.2%)	.542

Meals on Wheels^1^	T_0_	6 (7.0%)	3 (4.6%)	0	.145	-	3 (7.1%)	6 (3.7%)	.329
	T_2_	9 (9.9%)	8 (11.9%)	2 (3.6%)	.253		5 (11.1%)	14 (8.3%)	.562

Institutional short-term nursing^1^	T_0_	4 (4.7%)	0	5 (9.3%)	.135	A, B	4 (9.5%)	5 (3.1%)	.069
	T_2_	15 (16.5%)	15 (22.4%)	9 (16.4%)	.581		10 (22.2%)	29 (17.3%)	.445

Daycare^1^	T_0_	2 (2.3%)	0	1 (1.9%)	.481	-	0	3 (1.8%)	.376
	T_2_	7 (7.7%)	3 (4.5%)	3 (5.5%)	.687		5 (11.1%)	8 (4.8%)	.114

Day clinic^1^	T_0_	0	1 (1.5%)	2 (3.7%)	.206	-	2 (4.8%)	1 (0.6%)	.107
	T_2_	0	0	0	-		0	0	-

Outpatient physiotherapy^2^	T_0_	14 (16.3%)	3 (4.6%)	2 (3.7%)	**.013^I^**	-	7 (16.7%)	12 (7.4%)	.064
	T_2_	11 (12.1%)	5 (7.5%)	3 (5.5%)	.348		4 (8.9%)	15 (8.9%)	.993

Outpatient occupational therapy^2^	T_0_	2 (2.3%)	2 (3.1%)	1 (1.9%)	.908	-	2 (4.8%)	3 (1.8%)	.274
	T_2_	3 (3.3%)	1 (1.5%)	1 (1.8%)	.727		1 (2.2%)	4 (2.4%)	.950

* total 8 informal caregivers, who could be reached at follow-up, refused the interview at baseline

^1 ^Support and relief services for informal caregivers ^2 ^patient-centered non-drug therapy, but not dementia-specific

^I ^Overall model remains significant in the multivariate analysis: Chi²(3) = 16.664; p = .001, predictors: study arm (Wald (2) = 6.746; p = .034) and Barthel-Index (Wald (1) = 7.968; p = .005) are significant predictors.

## Discussion

In this study, the diagnostic and therapeutic procedures of general practitioners treating dementia patients were examined with respect to guidelines for the diagnosis and treatment of dementia. Moreover, the utilization of "caregiver support groups" and "informal caregiver counseling" recommended by the general practitioner at the start of the study was analyzed after 2 years. The utilization of other support services was also examined.

### Diagnostic procedures

The diagnostic behavior also corresponds in large measure to the dementia guidelines. Nearly all patients undergo physical-clinical and laboratory-diagnostic examination. Apparently, imaging procedures are performed only in certain cases - less than half of the patient underwent additional imaging examination. The diagnostic behavior of the general practitioners does not differ between pre- and post-training in the frequency of use of physical examination, imaging procedures and laboratory diagnostics. Referral to a specialist had been made in nearly two-thirds of patients who were diagnosed earlier, and in nearly half of the newly-diagnosed patients.

The rates found in our study of laboratory diagnostics and physical-clinical examination, which are to be performed in every patient with suspected dementia according to the guidelines, are similarly high as those reported by general practitioners in an international comparison [9,13]. However, they are much higher than reported in another German study [10]: 81.7% of the general practitioners performed laboratory diagnostics according to that study, 12.9% imaging procedures, 69.4% a physical-clinical examination. In an American study, by contrast, 99% of the general practitioners reported performing a clinical examination and ruling out other causes. Moreover 96% reported performing a cognitive screening test [43], whereby these are hypothetical results in the sense of intention, and probably overestimate the reality. It is also reported that general practitioners critically consider for each patient whether an imaging procedure is necessary or not [13].

Referral to a specialist was in our study realized more often in patients who were diagnosed earlier, than in newly-diagnosed patients. Part of this decrease can be explained by the fact that some general practitioners felt themselves more capable of diagnosing dementias after training. The remainder can probably be attributed to acute events, such as sudden deterioration of symptoms, in patients with already established diagnoses and that these motivated the general practitioner to refer the patient to a specialist. Riedel-Heller et al. [1] showed that about one-third of general practitioners prefer to consult a specialist in diagnosing dementia. Weyerer and Schäufele [44] report on a similar rate of involvement of specialists in the treatment process (28%). This decreases with increasing age of the patient.

### Therapy

The therapeutic behavior of the doctors is less stringently in conformity with guide-lines, or colored by the more critical position of the guidelines for general practitioners issued by the German Society of General Practice on treatment with anti-dementia medications. It must also be remembered that the contents of the guidelines have been further developed since the doctors were trained. On average, about one-third of the newly-diagnosed patients were treated with anti-dementia drugs specific for AD. In study Arms B and C, in which the doctors were trained in therapy, somewhat more patients were thus treated than in Arm A, but this difference did not attain statistical significance. Behavior which was explicitly not in conformity with guidelines occurred more often in Arm A, in which doctors were not trained. However, this is only a statistical trend, possibly due to a lack of power, so that a positive influence of training can only be assumed but not statistically proven. Drug therapeutic practice corresponds quite precisely to that reported in the literature, namely that one-third of the dementia patients receive anti-dementia therapy [15,17]. The results show, that to a certain extent train-ing of general practitioners can influence therapeutic behavior. Other reasons why the therapeutic behavior is not stringently in conformity with the guidelines have to be ex-plored in further research. Possible interpretation differences in available guidelines might be one reason.

### Support Services

Informal caregiver's utilization of support services which were explicitly recommended by their general practitioner increased in the intervention arms compared to the control group by four-fold for counseling and five-fold for support groups. This is noteworthy, since utilization on the part of the informal caregivers was voluntary. Apparently, recommendation of an offer by a general practitioner who is already known and considered competent has a demonstrable motivating effect. There are repeated reports in the literature that utilization of support services like support groups and caregiver counseling is low. In a representative survey of informal caregivers in Germany caring for patients with all kind of diseases leading to the need for care, the rate was 5% for support groups, 6% for caregiver counseling [45] and 16.2% for all informational possibilities [46]. Internationally, the utilization rates for caregiver counseling vary between 4.1% in Europe [46] and up to 13.7% in North America [47,48]. The utilization rates of caregiver support groups also differs considerably between Europe at 4.8% [46] and North America with up to 14.0% [47,48]. In our study, we could observe a marked increase in utilization, at least for counseling (69%) in national and international comparison by means of recommendation by the general practitioner and contact established by the counselors. With respect to support groups, the level could be increased five-fold to approximately 15%, so that the utilization is now more at the "North American" than the "European" level.

By contrast, utilization of further support and respite services remains at a low level, with only few exceptions - namely home nursing and institutional short-term nursing - even when these services are made more familiar by the counselor. Less than 10% of dementia patients on average receive non-drug therapies: outpatient physiotherapy and occupational therapy. Here the therapeutic necessity and/or lack of availability are probable reasons. The possible explanations for the mostly low utilization rates of caregiver services are different. On the one hand, the presence of a care level classification and thus reimbursement of at least part of the costs influence the utilization of care-related relief measures like home nursing and institutional short-term nursing in the German Health Care system. At the start of the study, 57% of the dementia patients enrolled had no formal care level assignment (Social Code Book XI) and thus no reimbursement possibility. The increased utilization of these two services in the 2-year observation period can be explained in that as the illness progresses, the average degree of independence decreased and therefore the percentage of patients assigned a care level increased. Thus reimbursement possibilities were available to more informal caregivers.

However, other services, which are usually free of charge or at a low cost to the informal caregivers, such as visiting voluntary helper services or care groups, were also only used by ca. 5% of the informal caregivers even at follow-up, although these services were widely available in the study region. There are multiple reasons: A large proportion of the family caregivers set themselves the task of managing care on their own. It promotes self esteem ("I can do it!"). Pressure of expectations by the family and/or social environment are also barriers for caregivers to accept relief services. With services like the voluntary helper service, in which a "stranger" comes into the home, it must be remembered that one must accept that someone will see the situation "in one's own four walls". Utilization of services which are conducted outside the home, such as daycare centers, is limited by the need to prepare the dementia patient to go out and bring him/her to the facility.

Utilization of other support and respite services is at a low level, which is however comparable to the international level. For example, in our study there was a utilization rate for home nursing (at follow-up) of 31.5% on average. This corresponds approximately to the 27.7% reported by Philip and Ghosh in the UK [49] or the 23.4% reported by Lamura et al. across different European countries [46]. US-American studies report rates between 22.9% [50] and 46.7% [48]. Another example is found in the average utilization rate of care groups at 3.8% in our study, which is very similar to the internationally-reported utilization rates of between 4% and 5.8% [47,48].

There is no difference in utilization of services at follow-up between the study arms. That means the visiting counselors' recommendations of further formal and information support, depending on the care situation, did not result in an increase in utilization of support or relief services - unlike systematic recommendation of services by the general practitioner. It must be remembered that two-thirds of the patients suffered only from mild dementia at the start of the study, so that a need for relief was subjectively not perceived by the informal caregivers. It is seen, however, that overcoming the barriers to utilization of support services in dementia cited above make considerable further effort necessary. It could be helpful if the general practitioner and caregiver counselors would work more closely together to present the services from two sides and especially to repeat the advantages of using these services in the individual case [51-54]. On top of that it has to be taken into account that some support offers lead to the feeling of subjective stress by the caregivers. Thus only offers which meet very closely with the individual needs will have subjective and objective success in the sense of relief for the caregiver [55].

There are no significant differences in utilization of the services between urban and rural residents. This can be positively evaluated since utilization differences would primarily indicate inadequate availability, most likely in rural areas, leading to lower utilization rates.

### Study Limitations

This is a cluster-randomized study, which means that randomization was at the general practice level. Patients in a single GP-practice were thus automatically all in the same study arm. Possibly, patients of one GP are more similar treated than patients between different GPs. The implications of this method were taken into account in the statistical evaluation by applying the intra-class-correction (ICC). Due to the study design, which defines the MMSE value as an inclusion criterion, it was not possible to check the frequency with which general practitioners use dementia screening tests to confirm the diagnosis.

The sample was drawn in Middle Franconia, a region with urban and rural character, in Germany. Therefore the data might be generalizable mainly for the German Health Care system. All patients were members of the AOK Bavaria - Health Insurer. This health insurance has members of all "layers" of the society but is not overproportional used by the upper layer. Of course it may be possible that rather the motivated GPs with the more motivated patients and caregivers were willing to participate in the study. Therefore it might be possible that for example the guideline adherence was overestimated.

As a result of age and diagnosis, there were a significant number of drop-outs of patients (and thus their informal caregivers) due to death or entry into a nursing home. Informal caregivers of patients in whom one of these two events occurred within 2 years, were no longer asked about their utilization of support services. So there is an obvious bias in Questions 2 and 3, since we know that informal caregivers of patients with such an event (drop-outs) had already utilized more services at the beginning of the study than informal caregivers who were still participating after 2 years. We illustrated this in the results section using home nursing as an example.

The observation period of two years of patients and families is relatively long for a field study and in comparison to other studies in health care research. However, with a chronic progressive disease like dementia it would be utterly important to know how the rate of utilization of help services and the therapeutic procedures develop in the course of the illness.

A strength of the study lies in its good data quality. There are no complete missing cases in the data collected from the doctors on diagnostics and therapy. In the utilization data, which refer to the follow-up time point, most of the missing values are attributable to death or entry into a nursing home - which are defined endpoints of the study. Missing values due to persons who could not be reached or study withdrawal are rare.

It must also be emphasized that the reality of care is reflected by a naturalistic study design. The doctors were free in their diagnostic and therapeutic behavior, despite training. Nevertheless they showed a procedure in conformity with the guidelines for the diagnosing of dementia. The freedom was also expressed in the therapeutic strategy. One third of the patients receive an anti-dementia drug, as is actually recommended in the guidelines. The proximity of the study to care reality and thus to the transferability of results into practice was also taken into account in the intervention. Unlike other research on family interventions [e. g. [26,29]], participation in the interventions suggested by the general practitioner was voluntary, they were simply an offer. Nonetheless, in the interventions group we achieved a utilization rate for caregiver counseling of 67%, which means than more than two-thirds of the caregivers agreed to direct personal contact with a family counselor, even though this was not required of them.

## Conclusions

General practitioners behave largely in conformity with guidelines in their diagnostic procedures in dementia patients. The therapeutic behavior with respect to prescription of anti-dementia drugs was in the majority less in accordance with guideline recommendations. However, there are some hints that behavior in conformity with guidelines could be promoted by means of training. Utilization of support services by informal caregivers remains low, as long as the informal caregivers are not intensively motivated to use such services. The role of the general practitioners as a motivator for informal caregivers is very significant, since the mediation of support services through the general practitioner led to a clear increase in utilization. For research, it is important to investigate the efficacy of further concepts in overcoming barriers to utilization among the informal caregivers of dementia patients.

## Competing interests

The sponsors have commissioned two academic research institutions with the scientific evaluation of the IDA project by giving unconditional research funds. A contract between the sponsors and academic researchers ensures that the latter have the full scientific responsibility and have the right to publish the results. Members of the sponsoring organizations closely cooperate in design and conduct of the project, but only the academic researchers have full access to all of the data in this study and take complete responsibility for the integrity of the data and the accuracy of the data analysis. CD, EG, MGS, RH and PMe are independent scientists who have received funding for this study as described above. CD received an honorarium for presentation of the IDA-study results at a public symposium from Eisai GmbH. Sponsoring organizations: SW is employee of the AOK Bavaria - Health insurer, JL is employee of the Federal Association of the AOK, HM is employee of Eisai GmbH, PMa is Head of Market Access of Pfizer Germany and SR is a former employee of Pfizer Germany. The IDA project was initiated and financed by the four partners with equal rights in conception, development and implementation: the AOK Bavaria - Health insurer and the Federal Association of the AOK - one of the largest statutory health insurances in Germany - and the research-based pharmaceutical companies Eisai and Pfizer. The aim of this public-private-partnership is to improve the care of dementia patients and the support of their informal care-givers.

## Authors' contributions

CD carried out the statistical procedures, interpretation of the data and wrote the manuscript. RH is principal statistical investigator, participated in the design of the study and gave advice concerning data analysis. EG is principal clinical investigator, participated in the design of the study and helped to draft the manuscript. PMe contributed to the data analysis by calculating cluster-effect adjusted measures. MGS carried out the data management and quality assurance of the data. JL planned and supervised the project and gave advice concerning structuring the manuscript. HM, PMa, SR planned and accompanied the research project and revised the manuscript. SW was the head of the local project team and revised the manuscript. All authors read and approved the final manuscript.

## Pre-publication history

The pre-publication history for this paper can be accessed here:

http://www.biomedcentral.com/1472-6963/10/314/prepub
